# Prevalence and characterization of Ice Nucleation Active (INA) bacteria from rainwater in Indonesia

**DOI:** 10.1186/s12866-022-02521-1

**Published:** 2022-04-27

**Authors:** Vivia Khosasih, Niko Prasetyo, Edi Sudianto, Diana Elizabeth Waturangi

**Affiliations:** 1 Faculty of Biotechnology, Atma Jaya Catholic University of Indonesia, Jalan Jenderal Sudirman, Jakarta, 12930 Indonesia; 2 Present Address: Institute of Biomedical Sciences, Academia Sinica, Taipei, Taiwan 115; 3 Department of Life Sciences, National Cheng Kung University, Tainan, Taiwan 701

**Keywords:** Bacterial ice-nuclei, Ice nucleation active bacteria, Rainwater, Air, Indonesia

## Abstract

**Background:**

Ice nucleation active (INA) bacteria are a group of microorganisms that can act as biological nucleator due to their ice nucleation protein property. Unfortunately, little is known about their prevalence and characteristics in tropical areas including Indonesia. Here, we monitor the presence of INA bacteria in rainwater and air samples collected from Jakarta, Tangerang and several areas in Western Java, Indonesia for one year. We further identify and characterize selected Class A of INA bacteria isolated from these areas.

**Results:**

Most of the INA bacteria were isolated from rainwater samples collected during March–August 2010, particularly from Jakarta, Bandung, and Tangerang. A total of 1,902 bacterial isolates were recovered from these area. We found a limited number of bacterial isolates from air sampling. From ice nucleation activity assays, 101 INA isolates were found active as ice nucleator at a temperature above -10 °C. A large majority (73 isolates) of them are classified as Class C (active below -8 °C), followed by Class A (26 isolates; active at -2 to -5 °C) and Class B (two isolates; active at -5 to -8 °C). We sequenced the 16S rRNA gene of 18 Class A INA isolates and identified 15 isolates as Enterobacteriaceae, while the remaining three as Pseudomonadaceae. The vast majority of our Class A INA isolates were likely *Pantoea* spp. with several isolates were deduced as either *Pseudomonas*, *Cronobacter*, and *Klebsiella*. We found that these 18 Class A INA isolates had acquired resistance to antibiotics erythromycin and ampicillin, which are considered two critically important antibiotics.

**Conclusions:**

Our results showed that the prevalence of INA bacterial population varies across locations and seasons. Furthermore, our isolates were dominated by Class A and C INA bacteria. This study also cautions regarding the spread of antibiotic resistance among INA bacteria.

**Supplementary Information:**

The online version contains supplementary material available at 10.1186/s12866-022-02521-1.

## Background

Ice nucleation active (INA) bacteria can catalyze heterogeneous ice crystallization at a temperature just below 0 °C [1, 2]. Repetitive octapeptide protein complex in their outer membrane, known as ice nucleation protein (INP), has been shown to induce the ice nucleation activity in *Pseudomonas* [3, 4]. INP promotes the positioning of water molecules into an ice-like structure, resulting in the phase transition from liquid (water) to solid (ice) [3, 5, 6]. INA bacteria are classified into three classes, Class A, Class B, and Class C, depending on their chemical structure and INP activity level [7–9].

INA bacteria are typically Gram-negative, epiphytic, and phytopathogenic [9]. They are abundant on plants and crops [10, 11], but it also had been isolated from clouds [4, 12], rain and snow [13, 14], and air [15], suggesting their role in the hydrological cycle [16]. Their presence and distribution are often linked to climate and atmospheric conditions [13, 17]. Many studies suggested their involvement in the initiation of precipitation [18–20], particularly in the snowfall and rainfall process [13, 14], by acting to promote cloud condensation and/or ice nuclei [21].

To date, most of the INA bacteria, particularly the well-studied *Pseudomonas syringae*, are isolated from the subtropical areas [22]. Their presence and distribution in tropical areas remain relatively limited. Unlike subtropical or temperate areas, the tropics have similar weather all year long and only have two seasons – dry and wet. The objectives of our study were to: (i) monitor if there is any seasonal/locational influence on the INA bacteria abundance in several areas in Indonesia and (ii) identify and characterize of INA bacteria isolates. Samples were collected from rainwater and air from several locations in Western Java, Jakarta, and Tangerang, Indonesia for one year and we identified the presence of INA bacteria in these environmental samples.

## Results

### Prevalence and distribution of INA bacteria isolates from rainwater and air

**Fig. 1:**
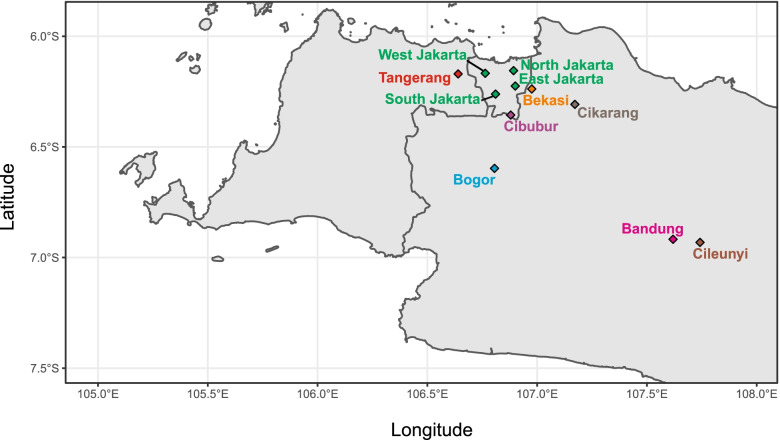
Map of sampling locations for the rainwater and air samples used in this study

**Fig. 2:**
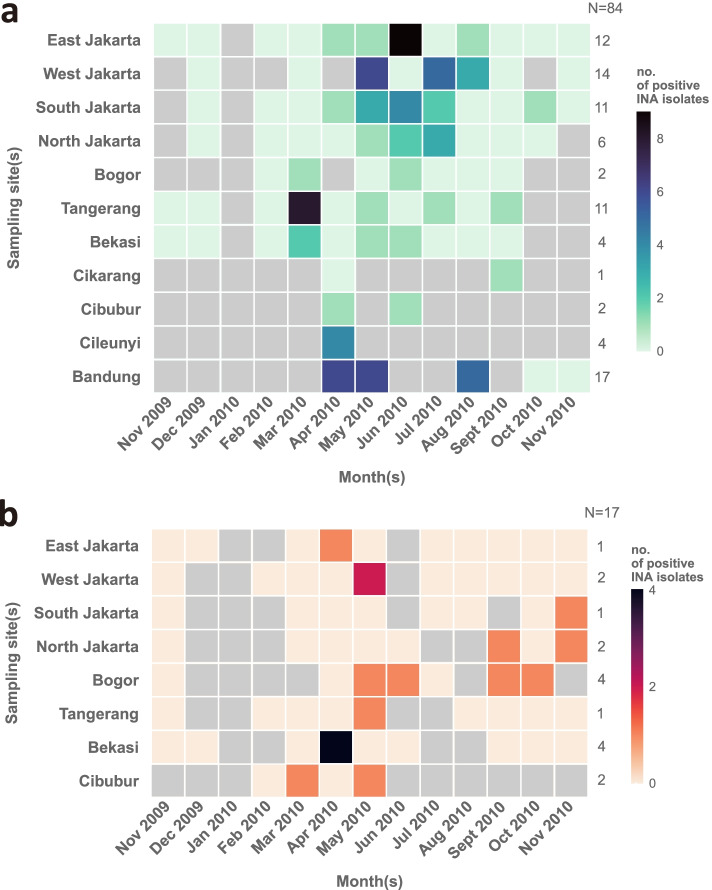
Heatmaps showing the spatial and temporal distribution of positive INA isolates from **a** rainwater and **b** air samples of several collection sites. Grey tile, data not available. N, number of positive INA isolates at each collection site

We collected rainwater (11 locations) and air samples (eight locations) from Western Java (including Bandung, Bekasi, Bogor, Cibubur, Cikarang, and Cileunyi) as well as Tangerang and Jakarta (four sites) (Fig. 1, Fig. 2). The total population size of bacteria isolated from rainwater varies from 10^4^ to 10^8^ cells/mL, while the total INA bacteria were 10 to 100 cells/mL (Table 1). Bekasi 2 showed the highest total population of both total bacteria and INA bacteria at 10^8^ and 10^2^ cells/mL, respectively, followed by South Jakarta 1, and West Jakarta 1 and 2. While for air samples, the population size of total and INA bacteria ranges from 10 to 100 cells/mL (Table 1). From our study we found that the highest total population of bacteria and INA bacteria were detected from air samples in Tangerang 2, followed by East Jakarta 1, South Jakarta 1, and West Jakarta 2. INA bacteria population was detected in at least one representative spot for each sampling location.

**Table 1:** Total bacterial and INA bacteria population recovered from environmental samples

Collection site	Total population {log [cells/ml(spread sample)]/[total/INA]}^a^	
	Rainwater samples	Air samples
East Jakarta 1	TNTC^b^/1.18	2.08/1.65
East Jakarta 2	4.69/1.40	TNTC/ND^c^
West Jakarta 1	7.36/1.21	1.87/ND
West Jakarta 2	7.84/1.15	1.79/1.48
South Jakarta 1	7.93/1.36	1.98/1.58
South Jakarta 2	6.44/1.09	2.25/ND
North Jakarta 1	TNTC/1.46	1.71/1.26
North Jakarta 2	TNTC/1.46	1.81/ND
Tangerang 1	6.33/1.07	1.92/ND
Tangerang 2	TNTC/1.60	2.09/1.93
Bekasi 1	TNTC/1.59	TNTC/1.41
Bekasi 2	8.13/1.78	1.28/0.95
Bogor 1	TNTC/1.83	1.90/1.04
Bogor 2	TNTC/1.48	1.88/ND

^a^Total population was estimated by dividing the log of the total bacterial population by the log of INA bacteria population

^b^TNTC, too numerous to count

^c^ND, not detected

Figure 2 showed the temporal and spatial distributions of the positive INA isolates from the rainwater and air samples. We found the number of INA bacteria from rainwater (84 isolates; Fig. 2a) were higher compared to air samples (17 isolates; Fig. 2b). Samples of rainwater from Bandung and Jakarta (especially West and East Jakarta), as well as Tangerang contained the highest number of positive INA isolates, while other sampling sites only had occasional positive of INA bacteria. In our study, we recovered the majority of INA bacteria during March–August 2010 than any other date. INA bacteria were rarely isolated from the air samples. Of the eight sampling sites, only Bekasi and Bogor had more than two positive INA isolates from the air samples. The remaining sampling sites only contributed one or two INA isolates. No clear patterns can be observed between collection dates and the number of positive INA isolates from the air samples.

### Ice nucleation protein classification and colony morphology

We recovered and purified 1,902 bacterial isolates that were further tested for ice nucleation activity. We found 101 INA isolates which were active at a temperature above -10 °C. We classified these isolates based on their ice nucleation temperature, including Class A (26 isolates), Class B (two isolates), and Class C (73 isolates). All of the Class A and B isolates were recovered from rainwater. The isolates from these two classes mostly form white or yellowish colony-color, translucent, and either mucoid or glossy-look colony (Additional file 1).

### Molecular identification of Class A INA bacteria

Here, we selected 18 Class A INA isolates (the most active INA bacteria) and sequenced their 16S rRNA gene to further identify to the genus and/or species level. Of the 18 Class A INA isolates, 11 isolates were similar to *Pantoea ananatis* strain 1846 with sequence similarity of 98–99%; two of the isolates were similar to *Pseudomonas lurida* strain DSM 15835 with sequence similarity of 97–100%; two isolates were similar to *Pa. agglomerans* strain DSM 3493 with 99% sequence similarity, and the remaining isolates showed high sequence similarity (98–99%) to *Ps. mosselii* strain CIP 105259, *Cronobacter sakazakii* strain ATCC 29544, and *Klebsiella pneumoniae* subsp. *rhinoscleromatis* strain R-70 (Table 2).

**Table 2:** Morphology and BLAST identification of 18 INA bacteria isolates assigned to Class A

Isolates	Collection site	Isolate morphology	Blast identification (Sequence similarity)	Accession number
Bdg/KB1356	Bandung	White yellowish-colony, translucent, mucoid	*Pseudomonas mosselii* strain CIP 105259 (99%)	JQ513917
Bdg/KB1357	Bandung	Yellowish-colony, translucent, mucoid	*Pantoea ananatis* strain 1846 (99%)	JQ513918
Bdg/KB1351	Bandung	Yellowish-colony, translucent, mucoid	*Pantoea ananatis* strain 1846 (99%)	JQ513919
Bdg/KB13511	Bandung	White yellowish-colony, translucent, mucoid	*Pantoea ananatis* strain 1846 (98%)	JQ513920
Bdg/KB13510	Bandung	Yellowish-colony, translucent, mucoid	*Pantoea ananatis* strain 1846 (99%)	JQ513921
JT1/KB1614	East Jakarta	Yellowish-colony, translucent, mucoid	*Cronobacter sakazakii* strain ATCC 29544 (98%)	JQ513922
Bdg2/KB1882	Bandung	Yellowish-colony, translucent, glossy-look	*Pantoea agglomerans* strain DSM 3493 (99%)	JQ513923
Bdg2/KB1888	Bandung	Yellowish-colony, translucent, glossy-look	*Pantoea ananatis* strain 1846 (99%)	JQ513924
Bdg2/KB1889	Bandung	Yellowish-colony, translucent, glossy-look	*Pantoea ananatis* strain 1846 (99%)	JQ513925
Bdg2/KB18811	Bandung	Yellowish-colony, translucent, glossy-look	*Pantoea ananatis* strain 1846 (99%)	JQ513926
Bdg2/KB1885	Bandung	Yellowish-colony, translucent, glossy-look	*Pantoea ananatis* strain 1846 (99%)	JQ513927
JB1/KB10512	West Jakarta	White-colony, translucent, mucoid	*Pantoea ananatis* strain 1846 (99%)	JQ513928
JB1/KB10511	West Jakarta	White-colony, translucent, mucoid	*Pantoea ananatis* strain 1846 (99%)	JQ513929
JB1/KB10510	West Jakarta	White-colony, translucent, mucoid	*Pseudomonas lurida* strain DSM 15835 (97%)	JQ513930
JT1/KB1610	East Jakarta	White yellowish-colony, translucent, mucoid	*Pantoea ananatis* strain 1846 (98%)	JQ513931
JT1/KB1611	East Jakarta	White yellowish-colony, translucent, mucoid	*Pseudomonas lurida* strain DSM 15835 (100%)	JQ513932
JT1/KB1615	East Jakarta	White yellowish-colony, translucent, mucoid	*Klebsiella pneumoniae* subsp. rhinoscleromatis strain R-70 (99%)	JQ513933
JB1/KB1876	West Jakarta	Yellowish-colony, translucent, mucoid	*Pantoea agglomerans* strain DSM 3493 (99%)	JQ513934

**Fig. 3:**
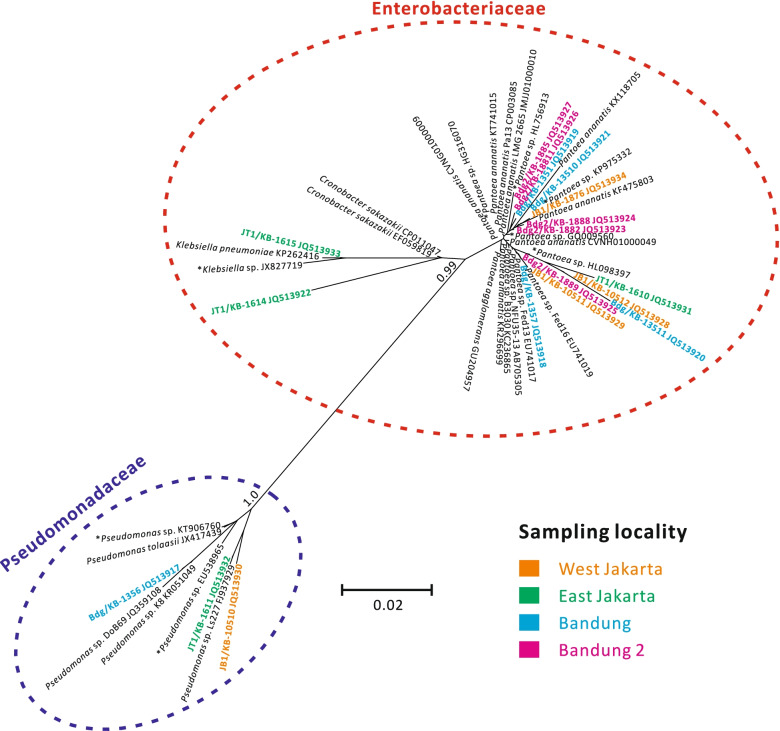
Unrooted phylogeny of 18 selected Class A INA bacteria isolates based on 16S rRNA gene. The collected strains were bolded and colored according to their sampling locality. Closely related reference sequences with the unclassified name were labeled with an asterisk (*) and named after their genera as identified by SILVA

The phylogenetic tree showed that these Class A INA bacteria were clustered into two families: Pseudomonadaceae and Enterobacteriaceae (Fig. 3). Fifteen of the isolates were classified as Enterobacteriaceae (mostly *Pantoea* spp.), while the other three isolates were likely *Pseudomonas* spp. (Fig. 3).

### Properties of Class A INA isolates

**Fig. 4:**
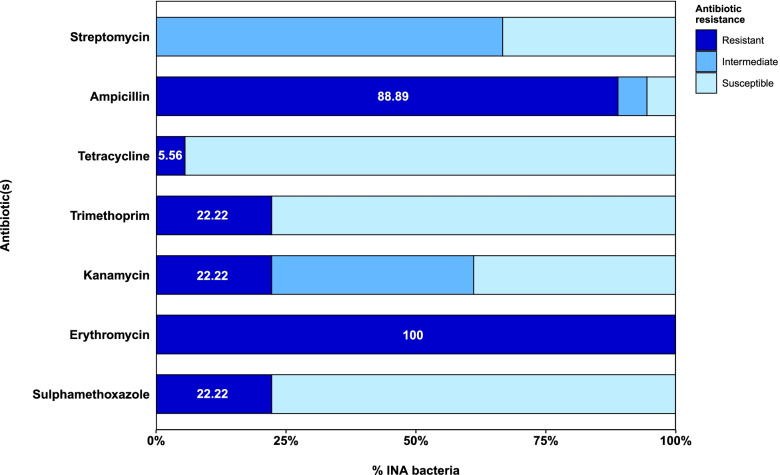
The antibiotic resistance of 18 selected Class A INA bacteria isolated from rainwater samples. The numbers in white font indicate the percentage of resistant INA isolates for each antibiotic

The 18 Class A INA isolates that we identified above were tested for antibiotic resistance properties. We found that all of the tested isolates were resistant to erythromycin (15 µg); sixteen of the isolates were resistant to ampicillin (10 µg); four were resistant to sulphamethoxazole (25 µg), kanamycin (30 µg), and trimethoprim (5 µg); and one was resistant to tetracycline (30 µg; Table 3; Fig. 4). None of the isolates was resistant to streptomycin (10 µg; Table 3; Fig. 4).

**Table 3:** Properties of 18 INA bacteria isolates assigned to Class A

Isolates	Antibiotic resistance							Biofilm formation	Anti-quorum sensing ability	EPS biosynthesis
	SMX	ERY	KAN	TMP	TET	AMP	STR			
Bdg/KB1356	R	R	S	R	S	R	I	-	-	+
Bdg/KB1357	S	R	S	S	S	R	S	-	-	-
Bdg/KB1351	S	R	I	S	S	R	S	-	-	-
Bdg/KB13511	R	R	R	R	S	R	I	+	-	+
Bdg/KB13510	S	R	S	S	S	R	S	-	-	-
JT1/KB1614	S	R	R	S	S	S	I	+	-	+
Bdg2/KB1882	S	R	R	S	S	R	I	-	-	-
Bdg2/KB1888	S	R	I	S	S	R	I	-	-	-
Bdg2/KB1889	S	R	I	S	S	R	I	-	-	-
Bdg2/KB18811	S	R	S	S	S	I	S	-	-	-
Bdg2/KB1885	S	R	I	S	S	R	I	-	-	-
JB1/KB10512	S	R	S	S	S	R	I	-	-	-
JB1/KB10511	S	R	I	S	S	R	I	-	-	-
JB1/KB10510	S	R	S	S	S	R	S	-	-	-
JT1/KB1610	R	R	S	R	S	R	S	+	-	-
JT1/KB1611	R	R	I	R	R	R	I	-	-	-
JT1/KB1615	S	R	I	S	S	R	I	+	-	+
JB1/KB1876	S	R	R	S	S	R	I	-	-	-

*Abbreviations*: *SMX* Sulphamethoxazole (25 µg), *ERY* Erythromycin (15 µg), *KAN* Kanamycin (30 µg), *TMP* Trimethoprim (5 µg), *TET* Tetracycline (30 µg), *AMP* Ampicillin (10 µg), *STR* Streptomycin (10 µg), *R* Resistant, *I* Intermediate, *S* Susceptible

Four out of 18 isolates (Bdg/KB13511, JT1/KB1610, JT1/KB1614, and JT1/KB1615) showed positive results from biofilm formation assay (Table 3). Likewise, only four out of 18 isolates (Bdg/KB1356, Bdg/KB13511, JT1/KB1614, and JT1/KB1615) produced Extracellular Polymeric Substances (EPS; Table 3). None of the 18 INA bacteria isolates showed anti-quorum sensing activity against wild-type of *Chromobacterium violaceum* (CV) and mutant *C. violaceum* 026 (CV 026; Table 3).

## Discussion

To the best of our knowledge, our study were the first to evaluate the prevalence and distribution of INA bacteria isolated from rainwater and air samples for a continuous period of at least one year in several location of western Java, Tangerang and Jakarta, Indonesia. Our study mainly focused on Jakarta and its surrounding urban areas. Jakarta as the capital city of Indonesia, is positioned on the northwest coast of Java Island on the embayment of the Java Sea. It lies on a low and flat terrain with an average elevation of 5 m above sea level and a temperature of 31–33 °C [23, 24]. It has a tropical monsoon climate with a rainy season from November–June [24].

The neighboring satellite towns, including Bogor, Tangerang, and Bekasi, have an average temperature of 26–28 °C [25–28]. Tangerang and Bekasi lie at < 50 m above sea level, while Bogor stands at a higher altitude of 190–330 m above sea level [29]. The rural areas of Cikarang and Cibubur, lie at 16–17 m above sea level, have an average temperature of 28–33.8 °C [30]. On the contrary, Bandung (and its neighboring Cileunyi), located about 140 km from Jakarta, is situated at 768 m above sea level [31]. Bandung has a cooler climate than Jakarta with a temperature of 23–24 °C [32].

Our data supports that INA bacteria are more commonly found in rainwater than air samples from the same collection site [15]. Constantinidou et al. [33] has previously described the movement of bacteria during rain due to the impact of raindrops, usually on leaves. Moreover, Graham et al. [34] observed an increase of viable bacteria concentration in aerosols during rain. Our findings indicate that INA bacteria were more frequently found from the rainwaters collected in March–August 2010. The number of INA bacteria from rainwater collected in November 2009–February 2010 and September–November 2010 were negligible (Fig. 2). A continuous multi-year observation would be of future interest to see if the trend persists.

Despite our infrequent sampling, Bandung showed the most positive INA bacteria of all our sampling sites – many of which were also classified as the most active Class A. This finding indicates that we were more likely to find INA bacteria in areas at higher altitudes with a cooler climate. A recent study emphasizes the importance of low temperature (ca. 5 °C) to activate the INP in *P. syringae* [35]. Thus, environmental condition of Bandung might be more favorable for the INA bacteria to survive and subsequently promote the INA bacteria abundance in the region.

Our results align well with a previous study that reported > 97% of INA bacteria as members of either Pseudomonadaceae or Enterobacteriaceae [20]. All of 18 sequenced Class A of INA isolates in this study were classified as members of these two families. The majority (13 isolates) were likely *Pantoea* spp., with three possible *Pseudomonas* spp.*,* and one each of *Cronobacter* and *Klebsiella*. Although *Pantoea* (Enterobacteriaceae) and *Pseudomonas* (Pseudomonadaceae) are widely known as INA bacteria [36–38], no studies have reported ice-nucleating capabilities in *Cronobacter* and *Klebsiella*. This might be the first record of *Cronobacter* and *Klebsiella* as Class A INA bacteria, as the most active type in INA classification. The identity of these two bacteria needs to be verified to assure that *Cronobacter* and *Klebsiella* are indeed capable to induce ice-nucleation.

It is interesting to note that the largest proportion (~ 72%) of INA bacteria recovered in Indonesia was those of the poorest ice nucleation activity. Class C of INA bacteria have fewer INP aggregates (5–10 INPs) than Class A of INA bacteria (> 30 INPs). These differences resulted in the dissimilar size of functional assembly at the bacterial outer cell membrane that arrange water molecules into an “ice-like” structure and nucleate ice formation. Large functional assembly of Class A INA bacteria is more effective at freezing water at a warmer temperature than the small functional assembly of Class C INA [39]. The Class C of INA bacteria may hold some of the previously unreported genus or species that has INA properties, such as *Stenotrophomonas* and Gram-positive *Lysinibacillus* collected from Virginia which were firstly reported to be able to nucleate ice at -8 °C [20]. Identifying these untapped resources may allow us to paint a better picture of the INA bacteria diversity in the tropics.

Although commonly known as plant pathogens, *Pantoea* (the most commonly found Class A INA bacteria in this study) has been increasingly reported as opportunistic pathogens for humans and developed several antibiotic resistance [40]. We tested the antibiotic resistance properties of 18 selected Class A of INA isolates and found that all of the isolates showed resistant to erythromycin and the vast majority (16 isolates) were resistant to ampicillin. On the other hand, they were susceptible to tetracycline (17 isolates), sulphamethoxazole (14 isolates), and trimethoprim (14 isolates). These results caution that both erythromycin and ampicillin resistance might be common among INA bacteria in Indonesia, especially in Jakarta. Although three other antibiotics were still effective, we also notice that the small prevalence of multidrug-resistant isolates, such as Bdg/KB13511 which was resistant to all antibiotics but tetracycline, or JT1/KB1611 that was intermediately resistant to kanamycin and streptomycin, but strongly resistant to any other antibiotics. These findings highlight the potential danger for the rise of multidrug-resistant of INA bacteria.

## Conclusions

In summary, our present study underlines the abundance of INA bacteria in Indonesia, especially from rainwater of Jakarta, Bandung, and Tangerang collected in March–August 2010. INA bacteria were rarely isolated from air samples. Class C INA bacteria constitute the majority (~ 72%) of our candidate INA isolates, followed by Class A (~ 26%) and Class B (~ 2%). We were able to identify several Class A INA isolates to the genus level, including *Pantoea, Pseudomonas, Cronobacter,* and *Klebsiella.* These Class A INA isolates were mostly resistant to erythromycin and ampicillin. Currently, our study was limited to several areas around Jakarta, Tangerang and Western Java, Indonesia. A broader sampling areas with diverse climate conditions across Indonesia and for a longer period of time (e.g. multi-year period) will be of our future interest. In addition, the identification of INA bacteria in our current study was constrained by the use of culture-based methods and may fail to identify microorganisms that are difficult to be cultured. The use of culture-independent metagenomics approach will be important to identify potential INA bacteria from the environmental samples that might have been missed by the traditional methods.

## Materials and methods

### Study area, climate, and sampling procedure

Rainwater and air samples were collected monthly over a yearly weather cycle starting from November 2009 to November 2010. Rainwater and air samples were collected from Bandung, Bogor, Bekasi, Cikarang, Cileunyi, Cikarang, Tangerang and from Jakarta (East Jakarta, West Jakarta, South Jakarta, North Jakarta) (Fig. 1), two spots (addresses) were chosen for each location. As a comparison, we collected rainwater samples from rural areas on the outskirts of Jakarta (Cikarang and Cibubur) and areas with a higher elevation than Jakarta (Bandung and Cileunyi) for at least one month (Fig. 2a).

#### Rainwater samples collection procedure

Samples of rainfall were collected in a sterilized bucket or washbasin at ground level for several hours for at least 2 L. Then, 50 mL of rainwater was transferred to a sterile vial for immediate laboratory processing. Rainwater samples were processed following Stephanie and Waturangi [15]. In brief, rainwater samples were diluted 2- to fourfold before spreading onto King’s B medium agar (in duplicates). Plates were incubated at 30 °C for 48 h, and total bacteria were counted. All bacteria colonies with different morphological characteristics were purified onto fresh King’s B medium agar for ice nucleation activity assay.

#### Air samples collection procedure

Ambient air were sampled and processed following Stephanie and Waturangi [15]. Luria Agar (LA) plates were exposed to the ambient air in each spot for 1 min. Plates were incubated at 30 °C for 48 h, and total bacteria were counted. All bacteria colonies with different morphological characteristics were purified onto fresh LA agar and tested for ice nucleation activity in a circulating alcohol bath. We used a 10 cm diameter of Petri dish plate.

### Ice nucleation activity and ice nucleation protein classification assay

#### Ice nucleation activity assay

The nucleation activity of INA bacteria was determined by the tube nucleation test following procedures described by Waturangi and Thjen [2] and Stephanie and Waturangi [15]. Briefly, each loop of representative colonies (~ 6 × 10^4^ CFU/mL) was suspended in 0.5 mL phosphate buffer (10 mM, pH 7.2). Then, 0.3 mL of the cell suspension was prepared in 3 mL phosphate buffer, and tested for the ice nucleation activity by immersing tubes in a circulating alcohol bath held at -10 °C. The freezing of phosphate buffer was taken as evidence of ice nucleation activity.

#### Ice nucleation protein classification

Ice nucleation protein was classified based on freezing temperature following procedures described by Stephanie and Waturangi [15]. The ice-nucleating activity of isolates was distinguished by measuring the warmest threshold of nucleation temperature at -2 to -10 °C in a circulating alcohol bath. Isolate that shows nucleation activity at a temperature between -2 and -5 °C is classified to Class A, isolate that is active at a temperature between -5 and -8 °C is classified to Class B, whilst isolate that has the activity at a temperature below -8 °C is assigned to Class C [7, 8].

### Genomic DNA extraction, PCR amplification of 16S rRNA gene, and DNA sequencing analysis

For INA bacteria identification, one loop of each 18 Class A INA isolates were extracted for the genomic DNA using the CTAB method described by Murray & Thompson [41] and used as the PCR template. We amplified and sequenced their 16S rRNA genes following procedures described in details by Stephanie and Waturangi [15]. Each PCR reaction was performed using 25 pmol of 63F/1387R primer pairs (New England BioLabs). DNA Amplification was performed using Perkin Elmer geneAmp PCR System 2400 with the following PCR conditions: initial denaturation at 94 °C for 2 min, 25 cycles of 94 °C for 30 s, 55 °C for 30 s, and 72 °C for 1 min, followed by a final elongation at 72 °C for 20 min. The following primers were used: forward, 5’-CAGGCCTAACACATGCAAGTC-3’; and reverse, 5’-GGGCGGAWGTGTACAAGGC-3’. PCR products were purified using PCR DNA Fragments Extraction Kit (Geneaid). DNA concentration was measured using Gene Quant (Amersham Biosciences), and then Sanger-sequenced using forward primer resulting in reads ranging from 200–650 bp. These DNA sequences were BLAST-searched against the non-redundant nucleotide (nr/nt) database with default parameters and submitted to GenBank (accession numbers: JQ513917–JQ513934).

### Sequence alignment and phylogenetic tree reconstruction

Sequences of the 18 isolated species were aligned and classified using SINA ACT (Alignment, Classification, and Tree) Service [42] in SILVA [43]. In the alignment step, unaligned bases at the ends were removed. We used 0.95 minimum identity for sequence query and limited the number of neighbors to two per query sequence. The 18 sequences along with 28 closely related sequences derived from the previous step were used to build the maximum likelihood (ML) tree using the FastTree option [44] with GTR + GAMMA model. The resulting tree was edited using FigTree (http://tree.bio.ed.ac.uk/software/figtree/).

### Properties of INA bacteria

Eighteen INA isolates assigned to Class A were tested for their bacterial properties. To characterize the properties of isolated bacteria, we performed antibiotic resistance analysis, biofilm formation assay, screening for anti-quorum sensing activity, and EPS biosynthesis assay.

#### Antibiotic resistance testing

The antibiotic resistance testing of selected INA isolates was performed using a disc diffusion test according to Kirby-Bauer [45]. INA isolates were streaked continuously onto King’s B agar, followed by placing antibiotic discs on the agar surface. These plates were then incubated at 30 °C for 48 h. The disk diffusion protocol and the interpretative criteria used were based on the Clinical and Laboratory Standards Institute (CLSI) guidelines standard [45]. Seven antibiotics discs used in this study were sulphamethoxazole (25 µg), erythromycin (15 µg), kanamycin (30 µg), trimethoprim (5 µg), tetracycline (30 µg), ampicillin (10 µg), and streptomycin (10 µg). All antibiotic discs were from Oxoid (Basingstoke, United Kingdom).

#### Biofilm formation assay

Each of 18 INA isolates was inoculated into Brain Heart Infusion Broth (BHIB; Oxoid, Basingstoke, United Kingdom), and incubated at 30 °C for 24 h. After incubation, the biofilm formation was assayed using protocol described by O’Toole and Kolter [46]. In brief, 1% of crystal violet solution was added to each reaction tube containing INA inoculate, followed by incubation at room temperature for 1 h. Then, the crystal violet dye was discarded, and reaction tube was rinsed thoroughly with deionized water. A positive result was shown through the formation of a violet ring in the reaction tube.

#### Screening for anti-quorum sensing activity

Anti-quorum sensing screening was performed according to McLean et al. [47]. Firstly, INA bacteria isolates were streaked continuously onto the LA plate (Oxoid, Basingstoke, United Kingdom) in a straight line. LA plates were then incubated at 30 °C for 48 h. The indicator strains, wild-type *Chromobacterium violaceum* (CV) and mutant *C. violaceum* 026 (CV 026), were inoculated separately in Luria Broth medium (Oxoid, Basingstoke, United Kingdom) at 30 °C for 24 h. Next, the semisolid agar of indicator strains was overlaid to the LA plates of INA isolates, followed by incubation at 30 °C for 48 h. The formation of clear zones showed the inhibition of violacein pigment production by the indicator strains.

#### EPS biosynthesis assay

The exopolymer-producing INA bacteria were screened by plating the selected INA isolates onto Tryptic Soy Agar (TSA; Oxoid, Basingstoke, United Kingdom). TSA plates were then incubated at 30 °C for 24 h. The bacteria were screened for their ability to synthesis EPS based on colony morphology indicated by mucoid phenotypes from colony growth.

## Supplementary Information

**Additional file 1.** Morphology of INA bacteria isolates classifiedto Class A and B

## Data Availability

The 16S rRNA genes sequenced in this study are deposited into the NCBI database with accession numbers: JQ513917–JQ513934.
