# Supplementary material for: Prevalence and characterization of Ice Nucleation Active (INA) bacteria from rainwater in Indonesia
Source: BMC Microbiol. 2022 Apr 27;22:116. doi: 10.1186/s12866-022-02521-1 (PMC9044597; doi:10.1186/s12866-022-02521-1)
Supplement: Supplementary file 1 — Additional file 1. Morphology of INA bacteria isolates classifiedto Class A and B [file 12866_2022_2521_MOESM1_ESM.docx]

Additional file 1. Morphology of INA bacteria isolates classified to Class A and B

| Class A (active at temperature of -4^o^C) | |
| --- | --- |
| Isolate code | Morphology |
| B1/KB2665 | Yellowish-colony, translucent, glossy-look |
| Bdg/KB1351 | Yellowish-colony, translucent, mucoid |
| Bdg/KB1355 | Yellowish-colony, translucent, mucoid |
| Bdg/KB1356 | White yellowish-colony, translucent, mucoid |
| Bdg/KB1357 | Yellowish-colony, translucent, mucoid |
| Bdg/KB13510 | Yellowish-colony, translucent, mucoid |
| Bdg/KB13511 | White yellowish-colony, translucent, mucoid |
| Bdg2/KB1882 | Yellowish-colony, translucent, glossy-look |
| Bdg2/KB1885 | Yellowish-colony, translucent, glossy-look |
| Bdg2/KB1888 | Yellowish-colony, translucent, glossy-look |
| Bdg2/KB1889 | Yellowish-colony, translucent, glossy-look |
| Bdg2/KB18811 | Yellowish-colony, translucent, glossy-look |
| Cileunyi/KB441 | White-colony, opaque, mucoid |
| Cileunyi/KB444 | White-colony, opaque, mucoid |
| Cileunyi/KB446 | White yellowish-colony, opaque, mucoid |
| JB1/KB10510 | White-colony, translucent, mucoid |
| JB1/KB10511 | White-colony, translucent, mucoid |
| JB1/KB10512 | White-colony, translucent, mucoid |
| JB1/KB1876 | Yellowish-colony, translucent, mucoid |
| JT1/KB165 | White yellowish-colony, translucent, mucoid |
| JT1/KB167 | White yellowish-colony, translucent, mucoid |
| JT1/KB168 | White yellowish-colony, translucent, mucoid |
| JT1/KB169 | White yellowish-colony, translucent, mucoid |
| JT1/KB1610 | White yellowish-colony, translucent, mucoid |
| JT1/KB1611 | White yellowish-colony, translucent, mucoid |
| JT1/KB1614 | Yellowish-colony, opaque, mucoid |
| Class B (active at temperature of -6.5^o^C) | |
| JS2/KB952 | Peach-color colony, translucent, mucoid |
| JS2/KB9510 | White-colony, translucent, mucoid |
